# Editorial: The emerging role of large language model chatbots in gastroenterology and digestive endoscopy

**DOI:** 10.3389/fmed.2026.1865795

**Published:** 2026-05-18

**Authors:** Antonietta Gerarda Gravina, Raffaele Pellegrino, Rocco Maurizio Zagari

**Affiliations:** 1 Department of Precision Medicine, University of Campania Luigi Vanvitelli, Naples, Italy; 2 Department of Medical Sciences and Surgery, University of Bologna, Bologna, Italy; 3 Gastroesophageal Diseases Unit, IRCCS Azienda Ospedaliero-Universitaria di Bologna, Bologna, Italy

**Keywords:** clinical decision support, digestive endoscopy, gastroenterology, generative artificial intelligence, hepatology, large language models, medical ethics

Generative artificial intelligence (AI) has rapidly expanded in medicine in recent years. Large language models (LLMs) have predominated in this landscape and, recently have become multimodal enabling the processing of not only text but also files including images within their reasoning frameworks (1). This development, particularly in fields such as gastroenterology and digestive endoscopy, has provided a substantial impetus to test their applications and operational performance (2–4).

In parallel with these insights into the potential of LLMs in medicine, attention has also been drawn to the ethical and medico-legal issues surrounding the application of these systems in clinical practice and medical research, prompting a cautious interpretation of LLM outputs, in particular when applied to patients, and encouraging the regulation of their use (1, 5).

Within this perspective, Irfan and Sirvent critically examined the role of LLMs from an ethical standpoint, recognizing their potential in gastroenterology for diagnostic and therapeutic support. However, they underlined significant concerns, including the errors that such systems may generate, namely artificial hallucinations, the environmental costs associated with LLMs, and more technical issues, such as the potential tendency of the system to privilege certain sources over others, the absence of clear determinism in responses, limited transparency regarding sources, and challenges related to privacy and medico-legal responsibility. The authors concluded that LLMs may be integrated into practice only in presence of transparent data updating, equitable accessibility, environmental sustainability, and global epistemic inclusion under appropriate human oversight.

Within this framework, this Research Topic serves as a catalyst for original studies, reviews, and perspectives on the application of LLMs in gastroenterology and digestive endoscopy.

## What has already been accomplished with LLMs in gastroenterology and hepatology?

Omar et al. sought to address this question by conducting a systematic review of studies assessing the applications of both natural language processing and emerging LLMs in gastroenterology and hepatology. They showed a marked surge of research interest toward GPT-3 and GPT-4 models, in particular in the years 2023–2024. The authors highlighted how ChatGPT, which was the dominant LLM within this specific research setting, has been employed for the generation of scientifically sound medical information, for example in providing counseling on *Helicobacter pylori* infection or in responding to frequently asked questions from patients in the field of hepatology, and for the definition of management pathways, such as formulating the management of patients with colorectal cancer or identifying appropriate endoscopic surveillance intervals. Finally, it has also been utilized to assist medical students in competitive examinations by supporting them in answering examination-style questions during their preparation.

El-Kassas et al. conducted a national study to assess the adoption of AI in hepatology practice and research in the Middle East and North Africa. The study included nearly 240 professional respondents and concluded that, although there is optimism regarding the use of AI, its actual utilization remains limited with significant readiness gaps.

## What new data have emerged from this Research Topic?

Qin et al. proposed a multimodal framework for digestive endoscopy, the Deep Gastrointestinal Diagnosis Network, that integrated both endoscopic frames and textual clinical data through LLM, thereby creating an architecture that combines multi scale feature fusion, guided attention mechanisms, domain adaptation and uncertainty estimation, to enhance lesion detection, segmentation and classification, yielding promising results.

On the other hands, Liu et al. developed two distinct machine learning models with the aim of predicting in-hospital mortality of patients admitted to intensive care for acute upper gastrointestinal bleeding. They identified a model with promising accuracy and highlighted prognostically relevant factors, including the use of vasoactive drugs, Glasgow Coma Scale score, AIMS65 score, use of anticoagulants, and peripheral oxygen saturation.

Wang et al. evaluated two LLMs, ChatGPT-4o and DeepSeek-R1, in the perioperative pharmacological management of patients with gastric carcinoma by developing approximately 20 questions based on simulations and real clinical cases. They showed that these tools may have good practical utility, notwithstanding substantial limitations concerning transparency, identification of the sources used by the LLM, and the completeness of their outputs.

Finally, Ye et al. reassessed several LLMs, namely ChatGPT-3.5, ChatGPT-4, and ChatGPT-4o, using 30 questions on *Helicobacter pylori* infection based on international guidelines, with the outputs evaluated by three gastroenterologists. ChatGPT-4o was the most accurate model, although ChatGPT-3.5 was the most consistent moded in the responses.

## What remains to be done? What problems continue to beset LLMs in this application and setting?

In response to the question of what unresolved limitations persist in the application of LLM in gastroenterology, hepatology, and digestive endoscopy, several assertions may be derived from the data produced within this Research Topic:

- The risk of artificial hallucinations and inaccuracies, particularly in complex conditions such as gastrointestinal bleeding, decompensated chronic liver disease, gastrointestinal neoplasms, and inflammatory bowel diseases, becomes an absolutely significant limitation;
- Variability of outputs with consequent reduced reliability, reproducibility, and absence of a deterministic input-output relationship;
- LLMs are not always fully “explainable,” which creates a lack of transparency regarding sources and hinders the mechanisms of clinical auditing;
- Multimodal integration, particularly in the evaluation of endoscopic frames, remains largely underexplored and lacks of validation;
- Issues related to uncertainty in clinical and legal responsibilities prevent clear considerations regarding potential medical errors attributable to LLMs;
- Organizational and cultural barriers in economically disadvantaged geographical regions;
- Environmental costs;
- Privacy and data security concerns.

## Conclusions

LLM chatbots are extremely promising tools in gastroenterology and digestive endoscopy, easy to employ and available at sustainable costs; however, substantial limitations remain. Their current use in medicine must be cautious, vigilant and alert to inaccuracies, with the necessity that the mind at the helm remains the human one. LLMs are stochastic tools that operate on probabilities rather than on a form of reasoning comparable to human deduction (6).
